# Bioprospecting Marine Fungi from the Plastisphere: Osteogenic and Antiviral Activities of Fungal Extracts

**DOI:** 10.3390/md23030115

**Published:** 2025-03-07

**Authors:** Matteo Florio Furno, Vincent Laizé, Irene Arduino, Giang Nam Pham, Federica Spina, Mohamed Mehiri, David Lembo, Paulo J. Gavaia, Giovanna Cristina Varese

**Affiliations:** 1 Department of Life Sciences and Systems Biology, University of Turin, Mycotheca Universitatis Taurinensis (MUT), Viale Mattioli 25, 10125 Turin, Italy; matteo.floriofurno@unito.it (M.F.F.); federica.spina@unito.it (F.S.); 2 Centre of Marine Sciences, University of Algarve, 8005-139 Faro, Portugal; vlaize@ualg.pt (V.L.); pgavaia@ualg.pt (P.J.G.); 3 Department of Clinical and Biological Sciences, University of Turin, Regione Gonzole, 10, Orbassano, 10043 Turin, Italy; irene.arduino@unito.it (I.A.); david.lembo@unito.it (D.L.); 4 Marine Natural Products Team, UMR 7272, Institut de Chimie de Nice, Université Côte d’Azur, CNRS, 06108 Nice, France; nam.phamgiang@phenikaa-uni.edu.vn (G.N.P.); mohamed.mehiri@univ-cotedazur.fr (M.M.)

**Keywords:** secondary metabolites, plastisphere, *Aspergillus jensenii*, SSF, SmF, microplastics

## Abstract

Marine microplastics (MPs) represent a novel ecological niche, populated by fungi with high potential for pharmaceutical discovery. This study explores the bioactivity of fungal strains isolated from MPs in Mediterranean sediments, focusing on their osteogenic and antiviral activities. Crude extracts prepared via solid-state and submerged-state fermentation were tested for their effects on extracellular matrix mineralization in vitro and bone growth in zebrafish larvae, and for their activity against the respiratory syncytial virus (RSV) and herpes simplex virus type 2 (HSV-2). Several extracts exhibited significant mineralogenic and osteogenic activities, with *Aspergillus jensenii* MUT6581 and *Cladosporium halotolerans* MUT6558 being the most performing ones. Antiviral assays identified extracts from *A. jensenii* MUT6581 and *Bjerkandera adusta* MUT6589 as effective against RSV and HSV-2 at different extents, with no cytotoxic effect. Although chemical profiling of *A. jensenii* MUT6581 extract led to the isolation of decumbenones A and B, they did not reproduce the observed bioactivities, suggesting the involvement of other active compounds or synergistic effects. These results highlight the plastisphere as a valuable resource for novel bioactive compounds and suggest the need for further fractionation and characterization to identify the molecules responsible for these promising activities.

## 1. Introduction

Marine fungi have evolved remarkable biodiversity and unique biological metabolic pathways to adapt to a range of ecological conditions, including extreme environments characterized by high pressure, low temperature, anoxia, and light limitation [1]. These adaptations came along with the synthesis of new metabolites, many of them yet to be discovered, that could present novel biological activities [2]. The antibiotic cephalosporin C was the first drug isolated from marine fungi in the 1950s [3]. Subsequently, more active metabolites were isolated from marine fungi [1,4,5]. The biochemical diversity of these metabolites is complex and rich, ranging from linear peptides and fatty acids to complex alkaloids, terpenes, and polyketides, which have shown antitumoral, antioxidant, antifungal, antibacterial, antiviral, and anticancer properties [1,2,6,7,8].

Among the various marine-associated habitats, sediments represent one of the most microorganism-rich environments. However, they are also highly competitive ecosystems, constantly challenged by factors such as nutrient limitation, pressure, anoxia, and the presence of toxic compounds. In the last decades, the persistent accumulation of microplastics (MPs) has emerged as a major concern [9]. The mycobiota associated with MPs in marine sediments of the Mediterranean Sea was recently investigated [10], leading to the identification of several yeast and filamentous fungal taxa. Furthermore, comparisons with the surrounding sediment-associated fungal communities revealed potential substrate specificity, underscoring the complexity of MP-associated fungal assemblages. Besides their ecological value, these MP-associated fungal strains could represent a valuable resource for innovative biochemical properties. In fact, MP-associated fungi have shown versatile metabolism, including the ability to degrade hydrocarbons, dyes, and xenobiotics (i.e., plastics), to produce biosurfactants (thus increasing the bioavailability of organic compounds), or being halotolerant and osmo-protective.

In this new human-made environment (i.e., the plastisphere), the competition for space and nutrients is very high, and the entire microbiome may be affected by this constant fight for survival. Although bacteria are known to produce bioactive compounds [11,12,13,14], marine fungi associated with the plastisphere could prevail by producing bioactive molecules involved in defense or antagonistic mechanisms. In fact, the ecological role of bioactive molecules has been associated with chemical defenses, where they act as potent inhibitors of physiological processes, promoting competition with other marine organisms to ensure their survival [15,16,17]. The biosynthetic pathways for secondary metabolites have evolved to produce chemical structures that specifically interact with biological targets. As a result, it can be inferred that many secondary metabolites possess inherent biological activities [18,19,20]. Therefore, bioprospecting plays a crucial role in deciphering the bioactive potential of these metabolites from marine microorganisms.

Although the ecological role of fungal secondary metabolites may be complex and not fully understood yet, the regulatory networks governing natural product production can be manipulated during fermentation processes to generate different bioactive compounds. Fermentation has been mainly classified into solid-state fermentation (SSF) and submerged fermentation (SmF) based on the type of substrate used. Studies have shown that SSF and SmF could activate different metabolic pathways, resulting in higher production of certain bioactive compounds in SSF, while SmF thrives on others [21,22,23]. Consequently, selecting the appropriate fermentation method is crucial, as it can significantly influence the outcomes.

The present study aims at investigating the MPs-associated marine fungi from Mediterranean sediments as possible sources of novel bioactive molecules of pharmaceutical interest, evaluating their potential in regenerative and infectivity medicine. Two different fermentation techniques, SSF and SmF, were evaluated for the production of bioactive molecules. Fungal crude extracts produced were screened using different assays for (1) osteoactive compounds using a fish bone-derived cell line capable of in vitro mineralization and the developing opercular bone of zebrafish larvae; and (2) compounds with antiviral activity against the respiratory syncytial virus (RSV) and herpes simplex virus type 2 (HSV-2).

## 2. Results and Discussion

Crude polar extracts of 15 marine fungi associated with the plastisphere of Mediterranean sediments (Table 1) were produced using SSF and SmF. In most cases, the productivity was 20–40-fold higher in SSF than in SmF. These differences in productivity across the two different methods are documented in the literature and attributed to the variations in fermentation techniques, which can trigger distinct metabolic responses [24,25]. However, in SSF conditions, the extract yield strongly depended on the fungus, ranging from 374.6 mg of *Vishniacozyma carnescens* MUT6591 down to 15.4 mg for *Aureobasidium pullulans* MUT6584. These differences were much less pronounced in SmF, where most extract yields were below 10 mg. The only outliers were *A. pullulans* MUT6584 and *Penicillium griseofulvum* MUT6588 (e.g., 12.0 mg and 108.2 mg, respectively). An average crude extract dry weight of 187.9 mg and 12.4 mg was achieved for SSF and SmF, respectively (Table 1).

### 2.1. Mineralogenic and Osteogenic Activities

#### 2.1.1. In Vitro Mineralogenic Assay

**Figure 1:**
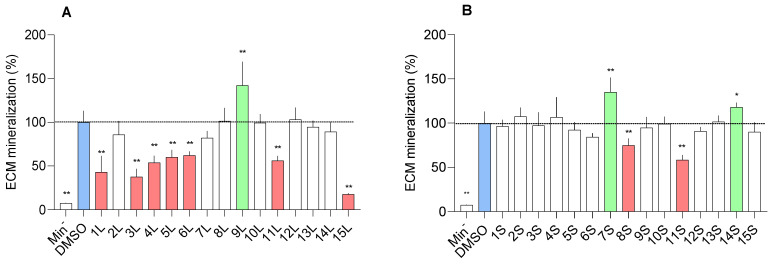
Mineralogenic effect of submerged fermentation (**A**) and solid-state fermentation (**B**) extracts assessed in VSa13 cells. All extracts were tested at 100 µg/mL, with the only exceptions of 9S and 15S that were tested at 10 µg/mL. Values are presented as mean ± standard deviation and as a percentage over the control group (DMSO, in blue). Normality was tested through Anderson–Darling test (*p* < 0.05). Asterisks indicate values significantly different (one-way ANOVA followed by Kruskal–Wallis test; * *p* < 0.001 and ** *p* < 0.0001). Each experimental group was tested against the control group (DMSO). Anti-mineralogenic extracts are represented in red, while pro-mineralogenic extracts are represented in green. Min^−^ indicates the control group not exposed to the mineralogenic cocktail n = 7.

Initial approaches for screening osteoactive compounds involved in vitro, ex vivo, and in vivo systems that have been developed and recently optimized [26,27,28]. In this study, the mineralogenic activity of extracts was assessed in vitro by testing the effect of the highest non-toxic concentration on extracellular matrix (ECM) mineralization of the gilthead seabream vertebra-derived cell line VSa13 for 17 days. The non-toxic concentration was determined by initially testing all extracts at 100 µg/mL and subsequently reducing the concentration to 10 µg/mL for extracts 9S and 15S when signs of cytotoxicity were observed. This stepwise approach ensured that the assessment of mineralogenic activity was conducted under non-toxic conditions. A total of 12 extracts exhibited mineralogenic activity (Figure 1). Among those, extracts 9L, 7S, and 14S increased ECM mineralization (42.17%, 35.16%, and 18.00% over the control, respectively) over the control, suggesting the presence of pro-mineralogenic compounds in these extracts. The osteoactive potential of marine fungi has already been assessed using in vitro cell systems [29,30,31,32,33]. Of special interest for this work, Marchese et al. [29] recently reported the osteogenic potential of the fungal community associated with the marine sea cucumber *Holothuria poli* in human mesenchymal stem cells. Among the 48 fungi tested in their study, *Penicillium chrysogenum* MUT1115 and *Penicillium citrinum* MUT1071 extracts promoted osteogenic differentiation, as demonstrated by increased alkaline phosphatase activity and ECM mineralization. Several compounds isolated from the marine fungi *Aspergillus jensenii*, *Sesquicillium microsporum*, and *Cladosporium pseudocladosporioides* were also tested in the same in vitro system [24,25,26,27], but none of them exhibited mineralogenic and/or osteogenic activity [34,35,36,37]. The present study instead identified 9L, 7S, and 14S as among the most active extracts, highlighting once again the crucial role of each single strain and the difficulties of generalizing the research findings on the whole species.

For some fungi, this study represents an outbreak in the field, being the first time that *Cystobasidium slooffiae, Aspergillus domesticus*, and *Parengydontium album* (extract 1-3-5) were identified as producers of compounds with osteogenic activity. However, it should be noted that these species have been poorly investigated in literature for their metabolic properties, in contrast to more studied genera, such as *Cladosporium* and *Penicillium.* No previous reports could be found also for *Cladosporium ramotenellum*, *Cladosporium halotolerans*, *C. pseudocladosporioides* (extract 6-8-14), *Bjerkandera adusta* (extract 4), and *P. griseofulvum* (extract 15). It is remarkable that despite the general interest in these taxa, osteogenic activity has never been reported before. *Cladosporium* species have been subjected to several metabolomic studies, and 244 (up to 286) compounds have been extracted [38,39], displaying mostly antimicrobial and cytotoxic activity [38]. Osteogenic bioactive molecules have not been identified yet.

As for *B. adusta*, despite its widely known capacity to degrade pollutants, has very little information available about its production of bioactive compounds. The synergistic interactions between natural phenolics and flavonoids in the *B. adusta* extract was directly correlated to a strong antimicrobial activity against pathogenic bacteria and yeast [40]. To the best of our knowledge, no other reports are currently available about other bioactive functions. A more complex scenario can be depicted for *Penicillium* spp. During 2021–2023 alone, more than 450 natural products from marine-derived *Penicillium* strains have been discovered [4], many of them showing cytotoxic, antiproliferative, and antimicrobial activity [41]. However, the involvement of *Penicillium*-derived molecules in osteoclastogenesis and osteogenesis is rare and was currently reported only for a few species, e.g., *Penicillium antarcticum* and *P. citrinum* [42,43], that do not include those investigated in the present study.

In this study, 9 extracts significantly reduced ECM mineralization when compared to the control. Notably, 7 of those were produced from SmF, a fermentation condition that may have triggered the production of compounds with anti-mineralogenic activity. These extracts should be further characterized to gain insights into the mechanisms and molecules responsible for this effect.

Interestingly, *A. pullulans* metabolome was insensitive to the growth conditions, since both SSF and SmF extracts (extract 11) exhibited anti-mineralogenic effects. This finding did not come as a surprise, as *A. pullulans* is known for its active metabolites, with many of them being used to set novel osteoporosis therapies. For instance, calcium polymalate (PMA-Ca) and beta-glucan polycan can enhance osteoblast proliferation, ECM mineralization, and osteogenic gene expression in vitro. In vivo, PMA-Ca can also promote bone growth and calcium conversion, while polycan improves bone density and reduces osteoporosis in mice [44,45,46]. However, Ca-PMA production in *A. pullulans* varies among strains and remains taxonomically unclear [47,48,49]. Some strains exhibit high Ca-PMA synthesis, while others lack characterization in this regard [50,51]. It can be hypothesized that the strain in this study may belong to a low or non-producing variety, explaining its anti-mineralogenic activity. Future genomic and metabolomic analyses should confirm this, helping to identify metabolites responsible for the anti-osteogenic effect.

#### 2.1.2. In Vivo Osteogenic Assay

**Figure 2:**
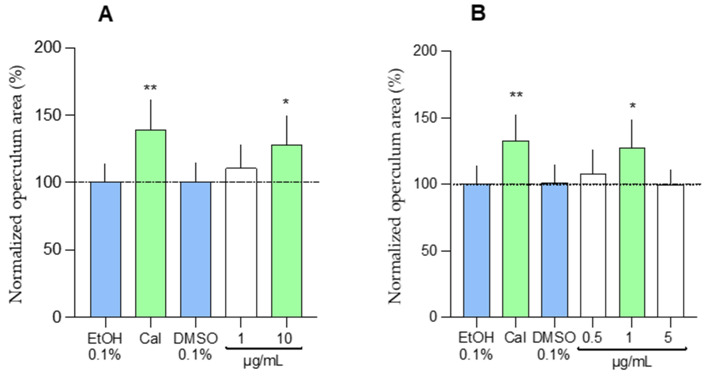
Osteogenic effect of extracts 8S (**A**) and 9L (**B**) assessed in the developing operculum of 6-dpf zebrafish larvae larvae through alizarin red S staining. DMSO and ethanol (EtOH) were used as negative controls for extracts and calcitriol (Cal), respectively. Changes in operculum area are expressed as percentages over the negative controls. Asterisks indicate values statistically different (one-way ANOVA followed by Dunnett’s multiple comparison test for extracts and DMSO, Student’s *t*-test for calcitriol and EtOH; * *p* < 0.001 and ** *p* < 0.0001) n = 15.

To assess the osteoactive effects of fungal extracts on a whole organism, 3-day post-fertilization (dpf) zebrafish larvae were exposed for 3 days to each extract, starting at 100 µg/mL (n = 7). In general, SSF extracts were found to be more toxic than SmF extracts (Figure S1). Consequently, only 4 SSF extracts were tested at 100 µg/mL, and the remaining 11 SSF extracts were tested at 10 µg/mL, while 9 SmF extracts were tested at 100 µg/mL, and the other 5 SmF extracts were tested at 10 µg/mL. Extract 9L exhibited the highest level of cytotoxicity and was tested at 1 µg/mL. As shown in Figure S1, extracts 8S, and 9L significantly increased the mineralized area of the opercular bone and were further tested at different concentrations and using a higher number of larvae (n = 15). Extract 8S and 9L increased the area of the operculum by 28.27% (at 10 µg/mL) and 27.62% (at 1 µg/mL), respectively (Figure 2).

A comparative analysis of in vitro and in vivo datasets identified extract 9L as both mineralogenic and osteogenic, highlighting the osteoactive potential of SmF extract from *A. jensenii*, which has never been reported in the literature before. Shin et al. isolated several secondary metabolites from *Aspergillus flocculosus* associated with the marine sponge *Stylissa* sp. [33]. Two of these metabolites, spertetranone D and wasabidienone E, weakly inhibited osteoclast differentiation, while a third metabolite, mactanamide, showed potent inhibition of osteoclast differentiation without any indications of cytotoxicity at effective concentrations. This compound was also isolated from a not-yet-described marine fungus of the genus *Aspergillus* associated with the brown seaweed *Sargassum* sp. [52] and could represent a taxon-specific metabolite. Future work should assess its presence in extract 9L and gain more insights into a possible role in osteoblast differentiation.

Extracts of 8S prepared from *C. halotolerans* exhibited a peculiar osteoactive effect characterized by an anti-mineralogenic activity in vitro and a pro-osteogenic activity in vivo. These opposite effects are per se intriguing, as one would expect a similar mineralogenic effect in vitro and in vivo. However, the extract could have different targets at cellular and organismal levels and affect ECM and bone mineralization in a direct or indirect manner. It is also possible that both activities originate from different molecules. Future studies should aim at evaluating all these hypotheses. Mohamed et al. have recently reviewed the secondary metabolites and the biotechnological potential of marine-derived *Cladosporium* species [38]. Among the nearly 300 compounds identified, none have been reported to have mineralogenic or osteogenic potential. However, the polyketide 7,4′-dihydroxy isoflavone (daidzein) isolated from the Antarctic fungus *Cladosporium* sp. NJF-6 was shown to decrease the expression of inflammatory markers like NF-kB, TGF β, TNF-α, IL-6, IL-8, and COX-2 using different systems both in vitro and in vivo [53], a result that may indicate a therapeutic potential to treat different diseases including osteoporosis [34,54]. The mineralogenic and osteogenic activities observed in fungal extracts, particularly those from *A. jensenii* MUT6581 and *C. halotolerans* MUT6558, suggest their potential involvement in key pathways regulating bone formation and extracellular matrix mineralization. Although the precise molecular mechanisms remain to be fully elucidated, we hypothesize that these extracts may influence osteoblast differentiation and activity through the WNT/β-catenin signaling pathway, which is essential for bone formation, or by modulating the RANK/RANKL/OPG axis, which governs osteoclast-mediated bone resorption [55,56,57]. The presence of bioactive secondary metabolites, such as polyketides or terpenoids, could underlie these effects, potentially acting as signaling modulators or enzyme inhibitors [58,59].

### 2.2. Antiviral Potential

The antiviral activity of the fungal extracts was assessed against RSV and HSV-2, important causes of diseases that can seriously impact human health [60,61,62,63,64,65]. These viruses were selected as prototypes for RNA and DNA viruses, respectively. In the first set of experiments, viral infectivity was assayed against cells exposed to increasing amounts of extracts to generate dose-response curves. Among the 30 extracts tested, 16 (53%) showed dose-dependent activity against RSV, with EC_50_ (half maximal effective concentration, or concentration of a compound that reduces viral infectivity by 50%) values ranging from 1.1 to 407.7 μg/mL (Table S1). Among those, extracts 4S, 4L, 9L, 15S, and 15L exhibited the best EC_50_ values (1.18–43.5 µg/mL). Similarly, 11 extracts (36.6%) significantly inhibited HSV-2 infectivity in a dose-dependent manner, exhibiting EC_50_ values ranging from 9.5 to 560.1 μg/mL, and only extracts 7S, 9S, and 9L resulted in EC_50_ values lower than 100 µg/mL (9.5–38.7 µg/mL). Extracts 4 and 9 of both SSF and SmF showed antiviral activities, suggesting a strain-specific production of antiviral molecule(s).

To assess the possibility that the observed antiviral activity may be due to the cytotoxicity of the extracts, cell viability was determined using the MTS assay. Several extracts demonstrated a high cytocompatibility, exhibiting CC_50_ (half-maximal cytotoxic concentration) values higher than 2000 µg/mL, while others exhibited some degree of toxicity at lower concentrations (Table S1). In particular, extracts 15L and 9S showed the lowest CC_50_ values on Hep-2 and Vero cells, respectively. It should be noted that cytotoxicity was not observed at the antiviral effective doses for all active extracts, indicating that the inhibitory activity was not due to cytotoxic effects.

While this study is the first report of antiviral activity for most of the fungi tested (12 out of 15), the presence of antiviral compounds in extracts prepared from *Cladosporium cladosporioides* (extract 2) and *P. griseofulvum* (extract 15) has already been documented [66,67]. Tang et al. showed that 4 compounds produced in the biotransformation of patchouli alcohol by *C. cladosporioides* exerted potent anti-influenza virus activity, with EC_50_ values in the range of 2.1–20.9 µM [68]. An inhibitor of the viral protease of the hepatitis C virus was also isolated from *P. griseofulvum*, showing an EC_50_ (concentration of a compound that inhibits viral infectivity by 50%) value of 3.8 µg/mL [68]. According to these previous observations, our work showed that extracts from both *C. cladosporioides* (extract 2) and *P. griseofulvum* (extract 15) exert inhibitory activity against another virus, i.e., RSV. This is the first time that an antiviral activity against RSV has been demonstrated for these two fungal strains. However, Yu et al. previously highlighted that the strain *Cladosporium* sp. WZ-2008-0042, associated with the gorgonian *Dichotella gemmacea*, produced a new pregnane (3α-hydroxy-7-ene-6,20-dione), which exhibited potent antiviral activity against RSV with an EC_50_ value of 0.12 mM [68]. Moreover, oxalierpenes A and B, produced by the mantis-shrimp-derived *Penicillium oxalicum*, showed antiviral activity against the H1N1 virus and RSV, with EC_50_ values ranging from 2.8 to 9.4 μM [69].

**Figure 3:**
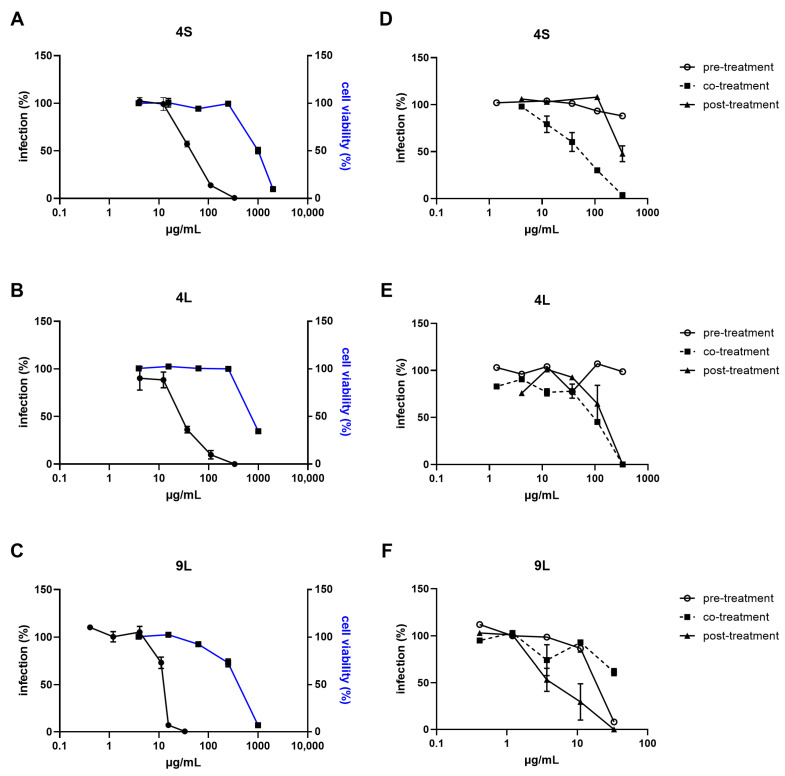
Antiviral activity of lead extracts 4S, 4L, and 9L against respiratory syncytial virus (RSV). (**A**–**C**) Antiviral activity was evaluated in cells infected with RSV and exposed to increasing concentrations of each extract. Virus infectivity (black) was assessed at 24 h post-infection, and cell viability (blue) was determined in the absence of viral inoculum. Both infection and viability were calculated by comparing extract-treated to DMSO-treated samples. (**D**–**F**) Time-of-addition assays were performed exposing cells to increasing concentrations of each extract for 2 h before infection (pre-treatment), for 2 h during infection (co-treatment), or for 24 h after removal of the virus inoculum (post-treatment). Virus infectivity was assessed at 24 h post-infection and was calculated by comparing extract-treated to DMSO-treated samples. Values are presented as mean ± standard error of the mean (SEM); n = 3.

**Figure 4:**
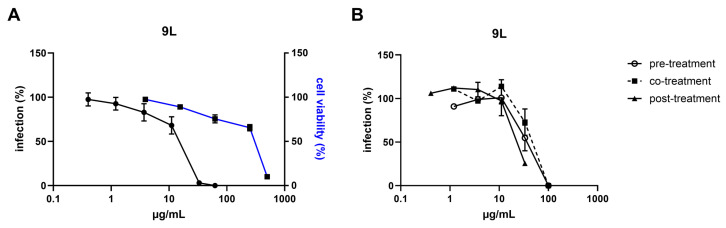
Antiviral activity of lead extract 9L against herpes simplex virus type 2 (HSV-2). (**A**) Antiviral activity was evaluated in cells infected with HSV-2 and exposed to increasing concentrations of the extract. Virus infectivity (black) was assessed at 24 h post-infection, and cell viability (blue) was determined in the absence of viral inoculum. Both infection and viability were calculated by comparing extract-treated and DMSO-treated samples. (**B**) Time-of-addition assays were performed in cells exposed to increasing concentrations of the extract for 2 h before infection (pre-treatment), for 2 h during infection (co-treatment), or for 24 h after removal of the virus inoculum (post-treatment). Virus infectivity was assessed at 24 h post-infection and calculated by comparing extract-treated and DMSO-treated samples. Values are presented as mean ± standard error of the mean (SEM); n = 3.

Extracts 4S, 4L, and 9L were selected, based on their high selectivity indices (SIs), to further characterize in vitro for their antiviral activity (Figure 3 and Figure 4). To gain insights into the mechanisms underlying the antiviral activity of these fungal extracts, time-of-addition assays were performed treating cells with the extract before, during, or after virus infection.

Results reported in Figure 3 show that extracts 4S and 4L inhibited RSV infectivity when added during the infection, with EC_50_ values similar to those obtained in the first antiviral assay (49.0–76.9 µg/mL). RSV replication was also partially inhibited by these two extracts when treatment was performed after the infection. Extract 9L was found to be active on both viruses and significantly inhibited RSV infectivity when added after infection (EC_50_ = 4.9 µg/mL) and, although to a lesser extent, before infection (EC_50_ = 17.9 µg/mL; Figure 3F). On the contrary, as reported in Figure 4, 9Ls ability to inhibit HSV-2 activity was observed independently of the time of exposure (i.e., it was effective when added before, during, and after infection), and EC_50_ values were in the same range as for the first antiviral assay (25.9–36.1 µg/mL; Figure 4B). The inhibitory activity herein observed at the different phases of virus replication may be ascribed to the well-known broad and diverse population of metabolites present in fungal extracts, which could account for the multiple targets of action of the extracts [70].

While this study reports for the first time antiviral activity in extracts prepared from *B. adusta* (extract 4) and *A. jensenii* (extract 9), other bioactivities have already been documented for fungi of the genera *Bjerkandera* and *Aspergillus*, e.g., antibacterial and antioxidant activity [40,71]. For instance, Georgousaki et al. also showed that benzoic acid derivatives isolated from *B. adusta* promoted the activity of two protein degradation systems, namely the ubiquitin-proteasome (UPP) and the autophagy-lysosome pathway [72]. Yi et al. recently reviewed the antiviral activity of compounds produced by marine microorganisms and highlighted the dominant position of species belonging to the genus *Aspergillus* as providers of antiviral natural products [73]. In this regard, Chen et al. recently reported an anti-RSV activity for compounds isolated from the gorgonian-associated *Aspergillus* sp. XS-20090B15 [74]. Xu-Hua Nong et al. also reported an anti-HSV type 1 (HSV-1) activity from territrem and butyrolactone derivatives purified from the marine fungus *Aspergillus terreus* SCSGAF0162 [75]. Among them, 12a-dehydroxyisoterreulactone A, arisugacin A, isobutyrolactone II, and aspernolide A demonstrated significant inhibitory effects, with EC_50_ values of 16.4, 6.34, 21.8, and 28.9 mg/mL, respectively [75]. Moreover, *Aspergillus* sp. SCSIO 41501, associated with gorgonians, produced aspergillipeptides D and E, which successfully inhibited HSV-1 with EC_50_ values of 9.5 and 19.8 μM, respectively; of note, aspergillipeptide D also demonstrated activity against an acyclovir-resistant strain of HSV-1 [76].

### 2.3. Extraction and Purification of Bioactives

**Figure 5:**
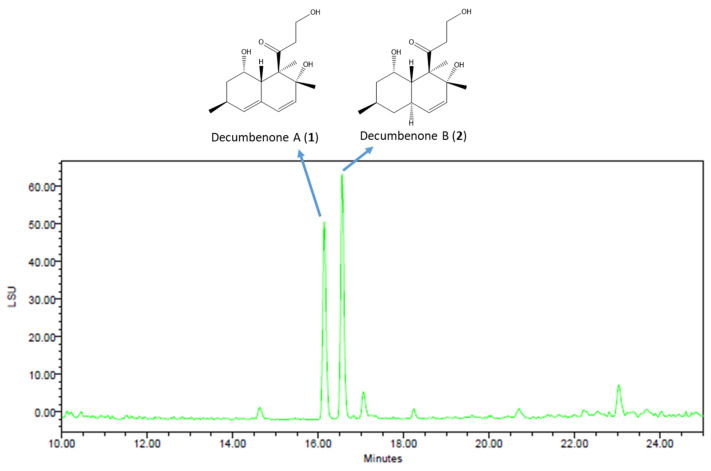
HPLC–ELSD chromatogram of *A. jensenii* extract 9L and the chemical structures of the two most abundant compounds: decumbenones A (**1**) and decumbenone B (**2**) (Column: NUCLEODUR Sphinx RP 250 × 4.6 mm; mobile phase: H_2_O-acetonitrile 90:10 for 5 min, then 90:10 to 0:100 for 25 min, containing 0.1% formic acid; flow rate: 1 mL.min^−1^; injection volume: 20 µL).

Since it exhibited promising pro-osteogenic and antiviral activities, the crude extract 9L was further analyzed by HPLC-UV/ELSD (Figure 5). Two compounds, predominant in the extract, were purified by HPLC and identified as decumbenones A and B by HRMS, NMR, and specific rotation values with literature (Figures S4–S10) [77].

Decumbenones are polyketides initially isolated from the terrestrial fungus *Penicillium decumbens* [77]; as shown in Table 2, they are also found in marine and terrestrial fungal species and have been assessed for several bioactivities.

Decumbenone A and B were described here for the first time as metabolites of the marine species of *A. jensenii*, even though they have already been found involved in many processes (Table 2). For instance, decumbenone A inhibited the melanization in *Magnaporthe grisea*, a rice blast pathogen [77], while decumbenones A and B selectively affected the human cancer esophageal cell line ECA109 with EC_50_ values of 12.41 and 15.60 µM, respectively [89]. In the present study, decumbenones A and B were hence evaluated for mineralogenic, osteogenic, and antiviral activities. However, neither of them replicated the observed bioactivities (e.g., mineralogenic, osteogenic, and antiviral) observed for the crude extract (Figures S2 and S3). This finding suggests that other compounds present in trace amounts or potential synergistic interactions among different metabolites could be responsible for the biological effects. Future studies should focus on a more comprehensive metabolomic approach, employing advanced techniques such as high-resolution mass spectrometry and tandem mass spectrometry to detect and characterize minor bioactive compounds. Additionally, fractionation strategies combined with bioassay-guided screening could help identify active metabolites that may work in synergy. Multi-omics approaches, integrating metabolomics with transcriptomics and proteomics, could provide insights into the regulatory pathways involved in the observed bioactivities. These methods would not only clarify the identity of the active compounds but also reveal possible mechanisms of action, paving the way for the development of novel therapeutic agents from marine fungal extracts.

## 3. Materials and Methods

The experimental setup of this study is summarized in Table 3, which provides an overview of the assays performed and their corresponding conditions.

### 3.1. Fungal Strains

A total of 15 fungal strains were isolated from MPs sampled in three areas of the Tyrrhenian Sea in Tuscany (Italy) in November 2019, as previously described by Florio Furno et al. [10]. Fungi were preserved at the *Mycotheca Universitatis Taurinensis* (MUT) of the University of Turin.

### 3.2. Growth of the Fungal Strains and Preparation of the Crude Extracts

Fifteen fungal strains were cultivated using SSF and SmF, following a protocol adapted from Yue et al. [90]. Briefly, fungi were pre-grown in 9 cm Petri dishes containing Malt Extract Agar (MEA) medium for 7–14 days at 24 °C. For both SSF and SmF, the inoculation was performed according to a modified Biolog protocol [91]. Whenever possible, a conidia suspension was prepared into a suspension medium (FF inoculation fluid, Biolog, Hayward, CA, USA), and the density, measured using a turbidimeter (Biolog, Hayward, CA, USA), was adjusted to the recommended value of 75%. The mycelium homogenate was prepared as follows: fungal biomass was collected from MEA plates with a sterile scalpel and homogenized using an UltraTurrax homogenizer (IKA, Staufen, Germany). The homogenate was transferred into the suspension medium using a sterile Pasteur pipette to achieve 40–60% transmittance.

For SmF, 3 mL of suspension was inoculated into 100 mL flasks containing 40 mL of Potato Dextrose Broth (PDB) for fermentation. After 2 weeks at 24 °C, the mycelium was separated from the broth by passing the fungal culture through a filter paper in a Buchner funnel under vacuum. In a separatory funnel, the broth was mixed twice with 100% ethyl acetate (EtOAc) at a proportion of 1:1 (*v*/*v*) to separate the aqueous phase and the organic phase and enhance the extraction yield. The organic phase was collected, and the solvent was removed using a rotary evaporator Re100-Pro (DLAB Scientific Inc., Beijing, China).

For SSF, 3 mL of suspension was inoculated into 100 mL flasks containing solid Rice Media (RM: 20 g of rice + 20 mL of mineral medium). The Mineral Medium (MM) was prepared as follows:10 mL/L of Mineral Solution (MS) and 1 mL/L of Trace Metal Solution (TMS). MS: 5 g of KCl, 5 g of MgSO_4_|7H_2_O, 0.1 g of FeSO_4_|7H_2_O in 100 mL of deionized water; TMS: 1 g of ZnSO_4_|7H_2_O and 0.5 g of CuSO_4_. After 2 weeks of cultivation at 24 °C, the fungus was homogenized using a sterile spatula and macerated in 100% EtOAc (1:1 *v*/*v*). After an overnight maceration, the organic phase was collected. The previous step was repeated with shorter incubation times (180 min). All the organic phases were pooled, and the solvent was removed using a rotary evaporator Re100-pro. Extracts were resuspended in 100% dimethyl sulfoxide (DMSO) and stored at 4 °C until used.

### 3.3. Assessment of Mineralogenic Activity

Gilthead seabream (*Sparus aurata*) bone-derived cell line VSa13 (Cellosaurus ID number CVCL_S952) [92] was maintained in Dulbecco’s modified Eagle medium (DMEM) supplemented with 10% fetal bovine serum (FBS), 1% penicillin–streptomycin, 1% L-glutamine, and 0.2% fungizone at 33 °C in a 10% CO_2_-humidified atmosphere [92,93]. Pre-confluent cell cultures were sub-cultured 1:4 twice a week using a trypsin–EDTA solution (0.2% trypsin, 1.1 mM EDTA, pH 7.4). Mineralization assays were conducted in 48-well NUNC plates seeded with 1.25 × 10^4^ cells per well and further incubated for 4 days until the culture reached confluency. The ECM mineralization was induced by supplementing culture medium with L-ascorbic acid (50 μg/mL), β-glycerophosphate (10 mM), and calcium chloride (4 mM). All extracts were initially tested at a concentration of 100 µg/mL. In case of cytotoxicity (e.g., cell death and altered morphology), extract concentration was lowered to 10 µg/mL and further evaluated. This stepwise approach allowed us to assess bioactivity while minimizing interference from toxic responses. Mineralizing cell cultures were exposed for 17 days to SmF or SSF extracts at the highest non-toxic concentration, or to 0.1% DMSO (vehicle) thereafter designed as the control group (CTRL). Cells cultured in non-supplemented medium (i.e., without mineralogenic cocktail and extracts) were also used as a negative control (Min^−^). Mineral deposition was assessed through alizarin red S (AR-S) staining and quantified by spectrophotometry as previously described [92].

### 3.4. Assessment of Osteogenic Activity

Zebrafish (*Danio rerio*) larvae at 3 dpf were exposed for 3 days to the fungal extracts as described by Tarasco et al. [94]. Briefly, freshly hatched 3-dpf larvae were transferred into 12-well plates at a density of 7 larvae per well; each well was filled with 5 mL of filtrated system water (i.e., water collected from the fish housing system) and supplemented with the extracts at the non-toxic concentrations or with 0.1% DMSO (vehicle/negative control sample). A group of larvae exposed to 10 pg/mL calcitriol dissolved in ethanol was used as positive control. Approximately 70% of the treatment was renewed daily. All the extracts were first tested at 100 μg/mL. Mortality and malformations were monitored for 3 days, and lower doses (10 or 1 μg/mL) were tested in case of severe toxic effects. After 3 days of exposure, larvae were given a lethal dose of anesthetic (MS-222 at 0.6 mM, pH 7.0, Merck KGaA, Darmstadt, Germania), washed in system water, and then stained with 0.01% of alizarin red S. Larvae were positioned laterally on a 2% agarose gel and then imaged using an MZ 7.5 fluorescence stereomicroscope (Leica, Wetzlar, Germany) equipped with a green light filter (λ_ex_ = 530–560 nm and λ_em_ = 580 nm) and a black-and-white F-View II camera (Olympus, Hamburg, Germany). The morphometric analysis of the operculum was performed using the fluorescence images and ZFBONE plugin for ImageJ [94]. The operculum area was corrected for interspecimen variability using the head area. After an initial screening, extracts exhibiting an osteogenic activity were retested using multiple concentrations and a higher number of larvae (n = 15), placed in a 6-well plate with 10 mL of system water per well.

### 3.5. Assessment of Antiviral Activity

Human epithelial cells (Hep-2; ATCC ID number CCL-23) and African green monkey fibroblastoid kidney cells (Vero; ATCC ID number CCL-81) were grown as monolayers in DMEM supplemented with 10% of FBS and 1% of antibiotic-antimycotic solution (Sigma-Aldrich, St. Louis, MO, USA). Cells were maintained at 37 °C in a 5% CO_2-_atmosphere. The RSV strain A2 (ATCC ID number VR-1540) and the HSV-2 strain MS (ATCC ID number VR-540) were propagated in Hep-2 and Vero cells, respectively. After propagation, viral progenies were clarified out of cell debris, aliquoted, and stored at −80 °C prior to virus titration by standard plaque assay.

All antiviral assays were performed in DMEM supplemented with 2% of FBS. For the antiviral assays, sub-confluent Hep-2 or Vero cells were seeded at a density of 1 × 10^4^ cells per well in 96-well plates and infected with a fixed inoculum of RSV or HSV-2 (multiplicities of infection—MOI of 0.01 or 0.005, respectively), while exposed to increasing amounts of extracts. After 24 h at 37 °C, cells were fixed in acetone:methanol (1:1), and infective events were visualized with an indirect immunocytochemistry procedure using specific primary antibodies directed against viral antigens, as described previously [95]. Infective events were microscopically counted, and viral infectivity was expressed as the mean % of infection ± standard error of the mean (SEM) of the extract-treated sample compared to the DMSO-treated sample (corresponding to 100% of infection).

Cell viability was measured using the MTS assay, as previously described [64]. Briefly, sub-confluent Hep-2 or Vero cells in 96-well plates were overlaid with serial dilutions of extracts and treated for the antiviral assay, without virus infection. The effect on cell viability of extracts was expressed as a mean % ± SEM, calculated by comparing the absorbances of treated cells with those of cells incubated with DMSO alone. Values of EC_50_, EC_90_ (concentration of a compound that reduces viral infectivity by 90%), CC_50,_ and relative 95% confidence intervals (CIs) were calculated. Where possible, selectivity indices of POMs were calculated as SI = CC_50_/EC_50_.

For time-of-addition assays that evaluate the virus replicative phase inhibited by a compound, sub-confluent Hep-2 or Vero cells were seeded at a density of 1 × 10^4^ cells per well in 96-well plates and incubated with increasing concentrations of extracts at 37 °C for 2 h before infection (pre-treatment), for 2 h during infection (co-treatment), or for 24 h after removal of the virus inoculum (post-treatment). Cells were concurrently infected with RSV or HSV-2 and treated for the antiviral assay.

### 3.6. Isolation of Secondary Metabolites from A. Jensenii Extract 9L

The culture broth (250 mL) of *A. jensenii* extract 9L was extracted as previously described. The crude extract (32.0 mg) was purified by HPLC (column: NUCLEODUR Sphinx RP 250 × 4.6 mm) using acetonitrile/water (25:75, 1 mL/min) containing 0.1% formic acid, to yield **1** (3.3 mg, Rt = 10.5 min) and **2** (2.1 mg, Rt = 12.3 min).

Decumbenone A (**1**): Yellow gum. [α]_D_^20^ + 35.0 (c 0.1, MeOH) (reported as [α]_D_^20^ + 54.0 (*c* 0.5, EtOH) [80]. Molecular formula: C_16_H_24_O_4_. ^1^H NMR (400 MHz, CD_3_OD) δ_H_ 5.93 (d, *J* = 9.9 Hz, H-11), 5.59 (s, H-9), 5.35 (d, *J* = 9.9 Hz, H-12), 4.22 (m, H-6), 3.83 (t, *J* = 6.2 Hz, H-1), 3.05 (dt, *J* = 18.0, 6.2 Hz, H-2a), 2.93 (q, *J* = 3.5 Hz, H-5), 2.85 (dt, *J* = 18.0, 6.4 Hz, H-2b), 2.52 (m, H-8), 1.86 (ddd, *J* = 9.9, 8.5, 4.2 Hz, H-7a), 1.46 (s, H-15), 1.24 (m, H-7b), 1.12 (s, H-14), 1.03 (d, *J* = 7.1 Hz, H-16). ^13^C NMR (400 MHz, CD_3_OD) δ_C_ 215.9 (C-3), 134.9 (C-12), 134.2 (C-9), 132.7 (C-10), 129.6 (C-11), 75.4 (C-13), 67.2 (C-6), 58.8 (C-1), 58.6 (C-4), 45.2 (C-2), 43.4 (C-5), 40.7 (C-7), 26.9 (C-14), 26.6 (C-8), 21.7 (C-16), 14.8 (C-15). HRESIMS *m/z* 303.1564 [M + Na]^+^ (calcd for C_16_H_24_O_4_Na^+^ 303.1567), 263.1640 [M − H_2_O + H]^+^ (calcd for C_16_H_23_O_3_^+^ 263.1642).

Decumbenone B (**2**): Yellow gum. [α]_D_^20^ + 10.0 (c 0.1, MeOH) (reported as [α]_D_^20^ + 8.0 (*c* 0.5, EtOH) [80]. Molecular formula: C_16_H_26_O_4_. ^1^H NMR (400 MHz, CD_3_OD) δ_H_ 5.39 (dd, *J* = 10.0, 1.8 Hz, H-11), 5.31 (dd, *J* = 10.0, 2.5 Hz, H-12), 4.09 (br s, H-6), 3.80 (td, *J* = 6.3, 2.8 Hz, H-1), 3.04 (dt, *J* = 18.3, 6.4 Hz, H-2a), 2.82 (dt, *J* = 18.3, 6.3 Hz, H-2b), 2.37 (m, H-10), 1.92 (dd, *J* = 10.7, 4.4 Hz, H-8), 1.82 (m, H-9a), 1.70 (m, H-7a), 1.52 (s, H-15), 1.15 (td, *J* = 13.5, 2.5 Hz, H-7b), 1.06 (s, H-14), 0.89 (d, *J* = 6.6 Hz, H-16), 0.73 (q, *J* = 12.2 Hz, H-9b). ^13^C NMR (400 MHz, CD_3_OD) δ_C_ 216.2 (C-3), 134.5 (C-12), 131.8 (C-11), 75.1 (C-13), 68.0 (C-6), 58.3 (C-1), 58.3 (C-4), 47.7 (C-5), 45.7 (C-2), 44.9 (C-7), 43.2 (C-9), 32.6 (C-10), 27.9 (C-14), 27.6 (C-8), 22.6 (C-16), 14.1 (C-15). HRESIMS *m/z* 305.1721 [M + Na]^+^ (calcd for C_16_H_26_O_4_Na^+^ 305.1724), 265.1797 [M − H_2_O + H]^+^ (calcd for C_16_H_25_O_3_^+^ 265.1799).

### 3.7. Statistical Analyses

Prism version 10.0 (GraphPad Software, Inc., La Jolla, CA, USA) was used for statistical analysis. For all the experiments, normality was tested with a D’Agostino-Pearson omnibus normality test or with an Anderson-Darling test (*p* < 0.05). Homoscedasticity was tested through the Brown-Forsythe test (*p* < 0.05). When the distribution of the data of all the experimental groups resulted normal and homogeneous, statistical differences between the control and the extracts were tested with a one-way ANOVA followed by Dunnett’s multiple comparison test (*p* < 0.05). If the distribution of the data of any of the experimental conditions resulted in non-normal or non-homogeneous statistical differences between the control and the extracts, they were tested with a non-parametric ANOVA test followed by Dunn’s multiple comparison test (*p* < 0.05). Sample sizes can be found in figure legends.

## 4. Conclusions

The bioprospection of the marine plastisphere has resulted in the identification of fungi able to produce different bioactive molecules with interesting pharmacological activities (i.e., osteogenic and antiviral). This work primarily underlines the hidden potential of marine fungi, with many species demonstrating bioactivities for the first time, supporting the importance of exploring the mycobiota associated with unique ecological niches such as the plastisphere. Moreover, it was demonstrated that fermentation techniques may affect the ability to activate distinct metabolic pathways. Both process yield and diversity of bioactive compounds could vary, highlighting the importance of optimizing cultivation strategies.

The extract prepared from *A. jensenii* has demonstrated promising pro-osteogenic and antiviral activities. Two abundant molecules, decumbenones A and B, were purified from this extract. However, none of these molecules exhibited the bioactivity found in the extract tested, both for osteogenic/mineralogenic and antiviral assays. The precise identity of the molecule or group of molecules responsible for the observed bioactivities remains unknown. As a result, it is imperative to conduct a comprehensive investigation into the compounds present in the extract, even those found in trace amounts, as these lesser-known constituents may play a significant role. Special consideration should be given to the possibility of synergistic effects, where the interaction between different molecules could amplify the bioactive properties. In the future, the systematic fractionation of the extract, followed by detailed validation of each bioactive fraction, will be critical for the successful identification and characterization of the specific molecules driving the reported biological effects.

## Figures and Tables

**Table 1:** List of fungi and crude extract dry weight associated with solid-state fermentation (SSF) and submerged-state fermentation (SmF).

Taxa	Id. Crude Extract SSF	Productivity for SSF (mg)	Id. Crude Extract SmF	Productivity for SmF (mg)
*Cystobasidium slooffiae* MUT6565	1S	121.9	1L	5.5
* Cladosporium cladosporioides * MUT6583	2S	188.8	2L	8.8
*Aspergillus domesticus* MUT6603	3S	131.7	3L	3.1
* Bjerkandera adusta * MUT6589	4S	43.0	4L	5.4
*Parengydontium album* MUT6579	5S	197.4	5L	4.1
* Cladosporium ramotenellum * MUT6556	6S	159.4	6L	2.7
*Sesquicillium microsporum* MUT6592	7S	37.4	7L	4.2
* Cladosporium halotolerans * MUT6558	8S	132.1	8L	3.8
* Aspergillus jensenii * MUT6581	9S	176.8	9L	9.0
* Sakaguchia dacryoidea * MUT6569	10S	355.3	10L	7.0
*Aureobasidium pullulans* MUT6584	11S	15.4	11L	12.0
*Vishniacozyma carnescens* MUT6591	12S	374.6	12L	6.9
* Kondoa aeria * MUT6563	13S	119.3	13L	3.4
* Cladosporium pseudocladosporioides * MUT6586	14S	132.2	14L	2.8
* Penicillium griseofulvum * MUT6588	15S	293.8	15L	108.2
Average productivity		187.9		12.4

**Table 2:** Decumbenones A and B: producing organisms and biological activities.

Decumbenone A					
Organism	Source	Bioactivity	Bioassay	Activity	Ref.
*Penicillium decumbens*	Terrestrial	Melanin inhibition	-	Active	[77]
*Aspergillus sulphureus*	Marine	Cytotoxicity Antiproliferation Antitumoral	Cancer cell lines (SK-MEL-28, SK-MEL-5, HCT 116)	Cytotoxic against SK-MEL-5, SK-MEL-28 Inhibitor for all Antitumoral on SK-MEL-5 and HCT 116	[78,79,80]
		Stimulators of development of agricultural plants	-	Stimulating effect on root growth of spring wheat	
*Aspergillus versicolor*	Marine	Antimicrobial	ATCC bacterial and yeasts	None	[81]
		Antioxidant	DPPH and FRAP	None	
*Aspergillus versicolor*	Marine	Cytotoxicity	A549, SKOV-3, SK-MEL-2, XF498, HTC15 cell lines	None	[82]
		Antibacterial	meticillin-resistent clinical strains	None	
*Aspergillus versicolor*	Marine	Antitumoral	TDP1 inhibition assay	None	[83]
*Aspergillus versicolor*	Marine	-	-	-	[84]
*Aspergillus carneus*	Marine	Cytotoxicity	MTT assay	None	[85]
		Antiradical activities	DPPH assay	None	
		Influence on fertilized sea urchin ovum	-	None	
*Craterellus odoratus*	Terrestrial	Glucocorticoid metabolism regulation	Inhibition of 11β-HSD1	None	[86]
		Cytotoxicity	Cancer cell lines	None	
Synthesized molecule	-	Cytotoxicity	Cancer cell lines	None	[87]
**Decumbenone B**					
*Penicillium decumbens*	Terrestrial	Melanin inhibition	-	Inactive	[77]
*Aspergillus sulphureus*	Marine	Cytotoxicity, Antiproliferation Antitumoral	Cancer cell lines (SK-MEL-28, SK-MEL-5, HCT 116)	Cytotoxicity against SK-MEL-5, SK-MEL-28,	[78,79,80]
				Antiproliferative for all	
				Antitumoral on SK-MEL-5 and HCT 116.	
		Stimulators of development of agricultural plants	-	Stimulatory growth effect on roots of buckwheat	
*Aspergillus versicolor*	marine	Antimicrobial	ATCC bacterial and yeasts	None	[81]
		Antioxidant	DPPH and FRAP	None	
*Aspergillus versicolor*	marine	Cytotoxicity agains human solid tumor cell lines	A549, SKOV-3, SK-MEL-2, XF498, HTC15 cell lines	None	[82]
		Antibacterial	20 meticillin-resistent clinical strains	None	
*Aspergillus versicolor*	Marine	Antitumoral	TDP1 inhibition assay	None	[83]
*Aspergillus versicolor*	Marine	-	-	-	[84]
*Aspergillus carneus*	Marine	Cytotoxicity	MTT assay	None	[85]
		Antiradical	DPPH assay	None	
		Influence on fertilized sea urchin ovum	-	None	
*Aspergillus flavus*	Marine	Antimicrobial	ATCC bacterial and yeasts	None	[88]
*Craterellus odoratus*	Terrestrial	Glucocorticoid metabolism regulation	Inhibition of 11β-HSD1	None	[86]
		Cytotoxicity	Cancer cell lines	None	

**Table 3:** Overview of the experimental design and conditions.

**15 Strains Recovered from Marine Microplastics**
Solid State Fermentation: 15 crude extracts
Submerged Fermentation: 15 crude extracts
**Mineralogenic and Osteogenic Activities**
In vitro mineralogic assay
Endpoint: extracellular matrix mineralization using gilthead seabream vertebra-derived cell line VSa13
Conditions: highest non-toxic concentration (100 µg/mL for most extracts; 10 µg/mL for extracts 9S and 15S)
In vivo osteogenic assay
Endpoint: mineralized operculum area using 6-day post-fertilization zebrafish larvae
Conditions: highest non-toxic concentration (1, 10, and 100 µg/mL depending on the extracts)
**Antiviral Activities Against RSV And HSV-2**
Endpoint #1: viral infectivity reduction assay, based on the number of infected cells exposed to increasing extract concentrations
Conditions: extracts from 0.1 to 1000 µg/mL covering all steps of infection
Endpoint #2: time-of-addition assay, exposing cells to extracts before, during, or after viral infection
Conditions: the most active extracts
**Identification of Bioactive Molecules**
Endpoint #1: HPLC-UV/ELSD analysis of the crude extract
Conditions: Extract of Aspergillus jensenii 9L with pro-osteogenic and antiviral activities
Endpoint #2: purification of predominant compounds in 9L extract by HPLC and evaluation of mineralogic, osteogenic, and antiviral activities

## Data Availability

Datasets generated during and/or analyzed during the current study are available from the corresponding author on reasonable request.

## Supplementary Materials

The following supporting information can be downloaded at: https://www.mdpi.com/article/10.3390/md23030115/s1, Figure S1: Osteogenic activity of Liquid (A) and Solid (B) extracts in zebrafish larvae; Figure S2: Antiviral activity of decumbenone A and B from extract *A. jensenii* 9L; Figure S3: Osteogenic activity of decumbenone A and B from extract *A. jensenii* 9L; Figure S4: HPLC-ELSD-UV/VIS chromatograms of the EtOAc extract of *A. jensenii*-9L; Figure S5: HRESIMS of 1; Figure S6: 1H NMR (400 MHz) spectrum of 1 in CD3OD; Figure S7: 13C NMR (100 MHz) spectrum of 1 in CD3OD; Figure S8: HRESIMS of 2; Figure S9: 1H NMR (400 MHz) spectrum of 2 in CD3OD; Figure S10: 13C NMR (100 MHz) spectrum of 2 in CD3OD; Table S1: Antiviral activity of extracts.

## Abbreviations

The following abbreviations are used in this manuscript:

MP	Microplastic
RSV	Respiratory Syncytial Virus
HSV-1	Herpes Simplex Virus Type 1
HSV-2	Herpes Simplex Virus Type 2
SSF	Solid State Fermentation
SmF	Submerged Fermentation
ECM	Extracellular Matrix
PMA-Ca	Calcium Polymalate
EtOH	Ethanol
SI	Selectivity Indices
MEA	Malt Extract Agar
PDB	Potato Dextrose Broth
MM	Mineral Medium
TMS	Trace Metal Solution
MS	Mineral Solution
EtOAc	Ethyl Acetate
RM	Rice Media
DMSO	Dimethylsulfoxide
AR-S	Alizarin Red S
EC_50_	Concentration of a compound that reduces viral infectivity by 50%
EC_90_	Concentration of a compound that reduces viral infectivity by 90%
CC_50_	Half maximal cytotoxic concentration
IC_50_	Concentration of a compound that inhibit viral infectivity by 50%
CI	Confidence Interval
dpf	Days Post-Fertilization
FBS	Fetal Bovine Serum
DMEM	Dulbecco’s Modified Eagle medium
