# Supplementary material for: Bioprospecting Marine Fungi from the Plastisphere: Osteogenic and Antiviral Activities of Fungal Extracts
Source: Mar Drugs. 2025 Mar 7;23(3):115. doi: 10.3390/md23030115 (PMC11944246; doi:10.3390/md23030115)
Supplement: Supplementary file 1 [file marinedrugs-23-00115-s001.zip › Figure S1.pdf]

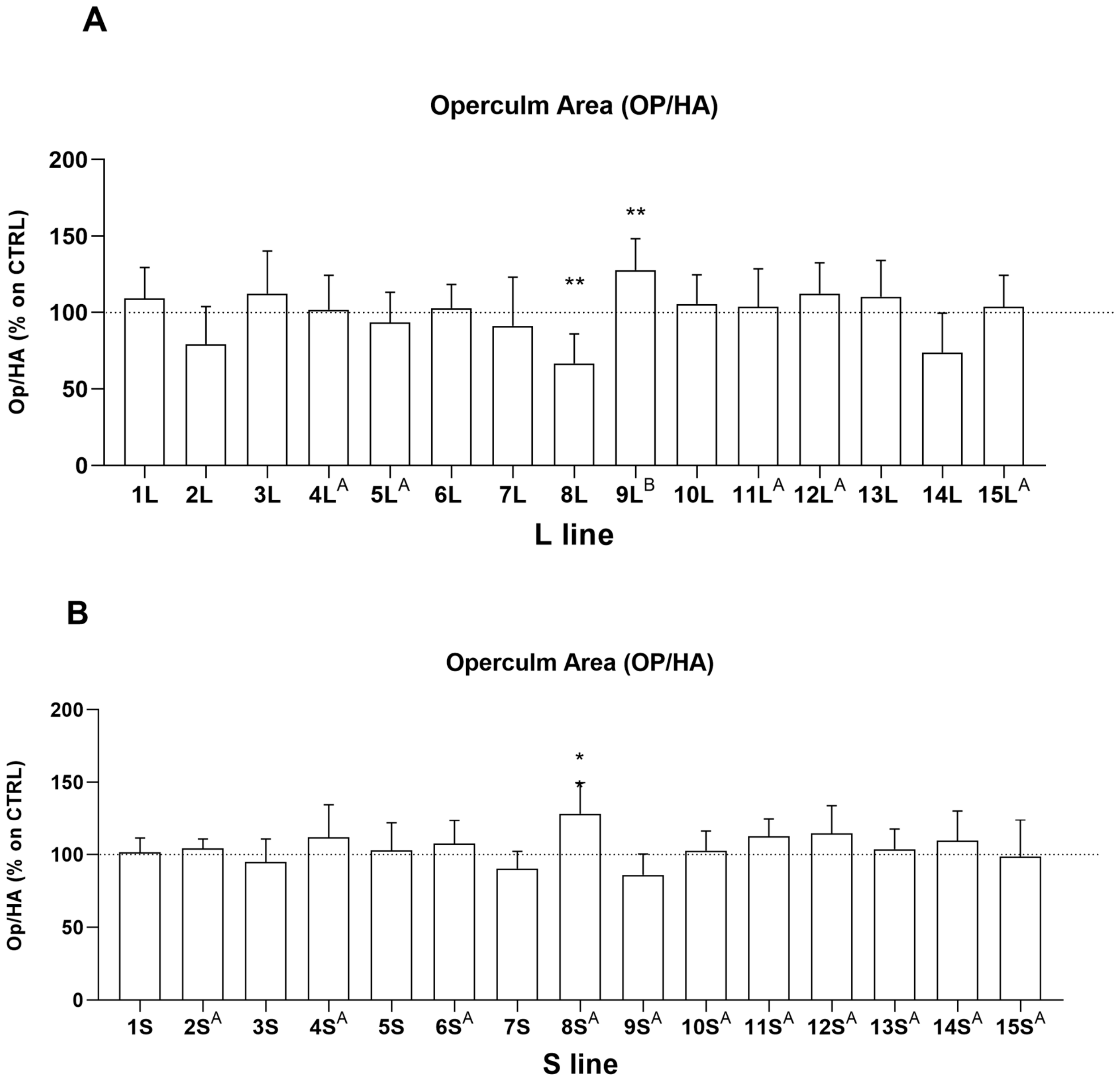

Figure S1. Osteogenic activity of Liquid (A) and Solid (B) extracts in zebrafish larvae. The normalized operculum areas (OpA/HA) are reported as percentage on the average of the control group (DMSO 0.1% v/v). The OpA/HA average of the control group for each extract tested is represented by the axis y =100 (%). Extracts were tested at 100 μg/mL, with the exception of ^{A} tested at 10 μg/mL and ^{B} tested at 1 μg/mL because of toxicity of the extracts. Statistical differences among the means are tested through One-way ANOVA (between control and each condition). p values are indicated as follow: * $p \leq 0.1$, ** $p \leq 0.01$; $n \geq 7$.
