# Supplementary material for: Bioprospecting Marine Fungi from the Plastisphere: Osteogenic and Antiviral Activities of Fungal Extracts
Source: Mar Drugs. 2025 Mar 7;23(3):115. doi: 10.3390/md23030115 (PMC11944246; doi:10.3390/md23030115)
Supplement: Supplementary file 1 [file marinedrugs-23-00115-s001.zip › Figure S2.pdf]

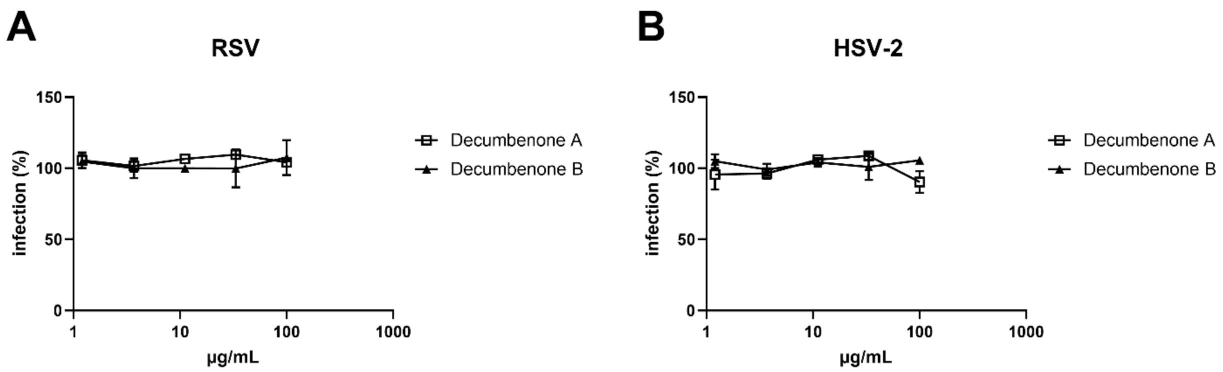

Figure S2. Antiviral activity of Decumbenone A and B from extract *A. jensenii* 9L. The anti-RSV (A) and anti-HSV-2 (B) activity was evaluated infecting cells in presence of increasing concentrations of the compounds, and assessing viral infectivity 24 h post-infection. The percentages of virus infection were calculated by comparing compound-treated and DMSO-treated samples. Results from two independent experiments are reported as the mean ± standard error of the mean (SEM).
