# Supplementary material for: Bioprospecting Marine Fungi from the Plastisphere: Osteogenic and Antiviral Activities of Fungal Extracts
Source: Mar Drugs. 2025 Mar 7;23(3):115. doi: 10.3390/md23030115 (PMC11944246; doi:10.3390/md23030115)
Supplement: Supplementary file 1 [file marinedrugs-23-00115-s001.zip › Figure S3.pdf]

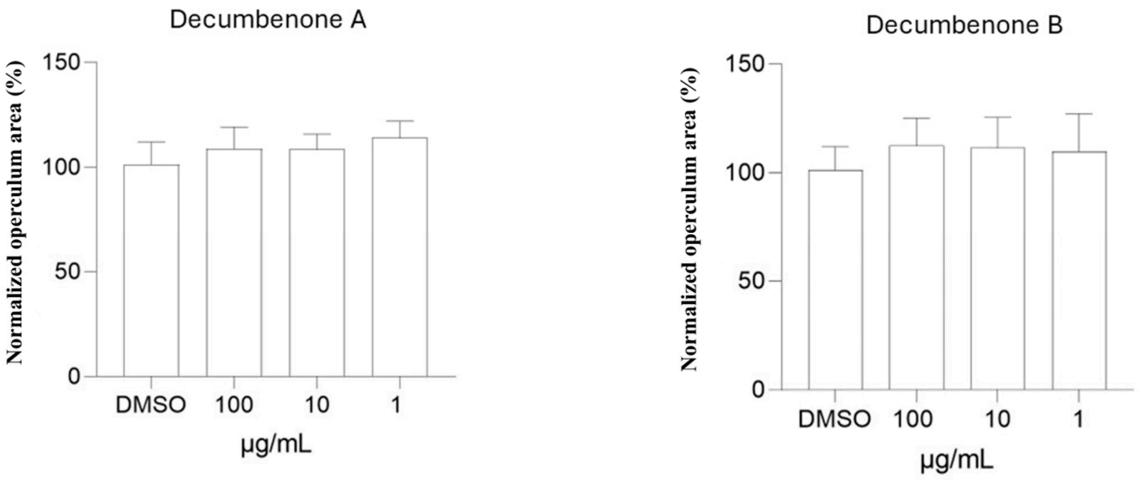

Figure S3. Osteogenic activity of Decumbenone A and B from extract *A. jensenii* 9L assessed in the developing operculum of 6-dpf zebrafish larvae through alizarin red S staining. DMSO was used as negative controls for extracts. Changes in operculum area are expressed as percentages over the negative controls
