# Supplementary material for: Bioprospecting Marine Fungi from the Plastisphere: Osteogenic and Antiviral Activities of Fungal Extracts
Source: Mar Drugs. 2025 Mar 7;23(3):115. doi: 10.3390/md23030115 (PMC11944246; doi:10.3390/md23030115)
Supplement: Supplementary file 1 [file marinedrugs-23-00115-s001.zip › Figure S4.pdf]

Sample Name:	6581L	Acquired By:	System
Sample Type:	Unknown	Sample Set Name:	Pham fungi Turin
Vial:	104	Acq. Method Set:	Alliance 2 PNM AN
Injection Volume:	20.00 ul	Run Time:	60.0 Minutes

Stacked Chromatograms

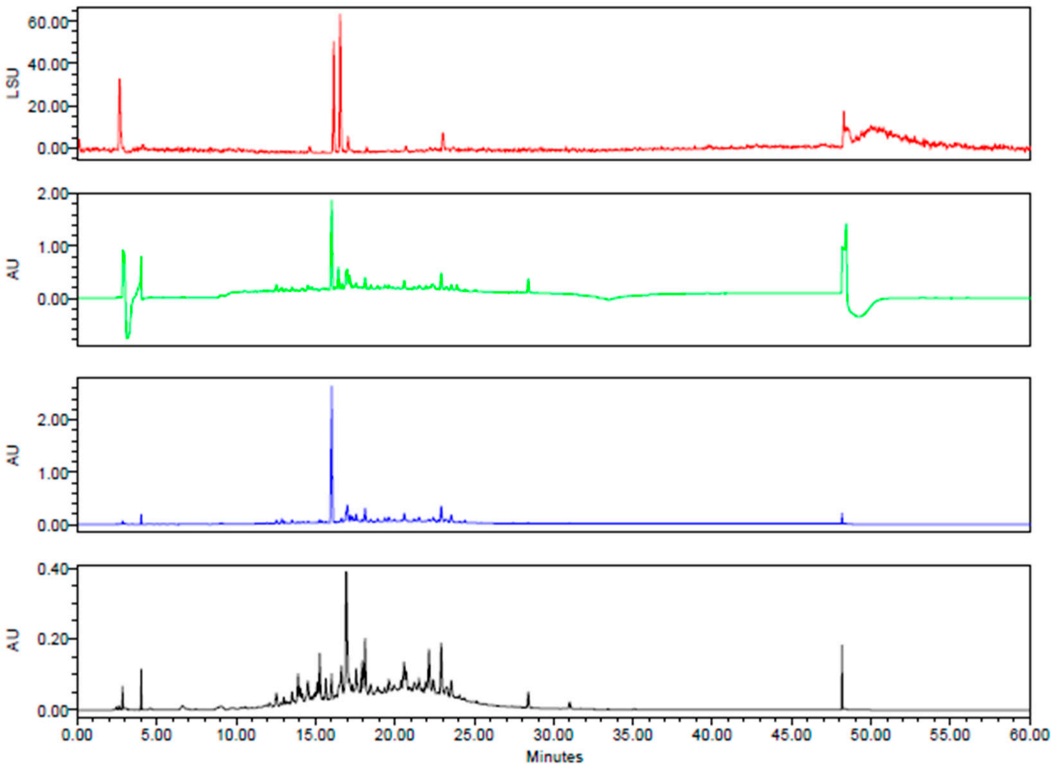

Vial 104; Injection 1; Date Acquired 2/17/2023 1:55:12 PM CET; Channel Name ELSD; Label ; SampleName 6581L

Vial 104; Injection 1; Date Acquired 2/17/2023 1:55:12 PM CET; Channel Name 214 nm; Label ; SampleName 6581L

Vial 104; Injection 1; Date Acquired 2/17/2023 1:55:12 PM CET; Channel Name 254 nm; Label ; SampleName 6581L

Vial 104; Injection 1; Date Acquired 2/17/2023 1:55:12 PM CET; Channel Name 280 nm; Label ; SampleName 6581L

Figure S4. HPLC-ELSD-UV/VIS chromatograms of the EtOAc extract of *A. jensenii*-9L.
