# Supplementary material for: Bioprospecting Marine Fungi from the Plastisphere: Osteogenic and Antiviral Activities of Fungal Extracts
Source: Mar Drugs. 2025 Mar 7;23(3):115. doi: 10.3390/md23030115 (PMC11944246; doi:10.3390/md23030115)
Supplement: Supplementary file 1 [file marinedrugs-23-00115-s001.zip › Table S1.pdf]

Table S1. Antiviral activity of extracts.

Compounds	RSV			HSV-2		
	EC_{50}*	CC_{50}§	SI#	EC_{50}	CC_{50}	SI
**1S**	n.a.	n.t.	n.a.	n.a.	n.t.	n.a.
**1L**	309.7	>2000	>6.5	n.a.	n.t.	n.a.
**2S**	169.4	902.1	5.3	n.a.	n.t.	n.a.
**2L**	n.a.	n.t.	n.a.	118.2	738.0	6.2
**3S**	327.2	>2000	>6.1	n.a.	n.t.	n.a.
**3L**	304.4	>2000	>6.6	444.2	>2000	>4.5
**4S**	43.5	1020	23.4	222.3	648.0	2.9
**4L**	29.1	855.5	29.4	101.1	560.7	5.5
**5S**	n.a.	n.t.	n.a.	105.5	221.6	2.1
**5L**	n.a.	n.t.	n.a.	n.a.	n.t.	n.a.
**6S**	n.a.	n.t.	n.a.	n.a.	n.t.	n.a.
**6L**	234.8	1959	8.3	560.1	>2000	>3.6
**7S**	n.a.	n.t.	n.a.	38.7	247.1	6.4
**7L**	n.a.	n.t.	n.a.	n.a.	n.t.	n.a.
**8S**	n.a.	n.t.	n.a.	n.a.	n.t.	n.a.
**8L**	n.a.	n.t.	n.a.	n.a.	n.t.	n.a.
**9S**	104.4	653.1	6.3	9.5	66.7	7.0
**9L**	12.2	317.2	26	12.1	169.3	14.0
**10S**	n.a.	n.t.	n.a.	n.a.	n.t.	n.a.
**10L**	n.a.	n.t.	n.a.	333.7	1983	5.9
**11S**	n.a.	n.t.	n.a.	n.a.	n.t.	n.a.
**11L**	n.a.	n.t.	n.a.	n.a.	n.t.	n.a.
**12S**	407.7	>2000	>4.9	173.5	1141	6.6
**12L**	330.3	>2000	>6.1	n.a.	n.t.	n.a.
**13S**	212.9	>2000	>9.4	n.a.	n.t.	n.a.
**13L**	303.8	>2000	>6.6	n.a.	n.t.	n.a.
**14S**	n.a.	n.t.	n.a.	n.a.	n.t.	n.a.
**14L**	358.8	>2000	5.6	n.a.	n.t.	n.a.
**15S**	19.7	271.4	13.8	n.a.	n.t.	n.a.
**15L**	1.18	13.9	11.8	n.a.	n.t.	n.a.

**Ribavirin**	7.9	>2000	>253.2	n.t.	n.t.	n.t.
**Acyclovir**	n.t.	n.t.	n.t.	0.37	>2000	>5405

* EC_{50}: half maximal effective concentration (concentration of compound expressed in μg/mL for all extracts, in μM for antiviral compounds used as control);
§ CC_{50}: half maximal cytotoxic concentration;
# SI: selectivity index ($SI = CC_{50}/EC_{50}$);
n.a.: not assessable;
n.t.: not tested.
