# Supplementary figures and images for: Bioprospecting Marine Fungi from the Plastisphere: Osteogenic and Antiviral Activities of Fungal Extracts
Source: Mar Drugs. 2025 Mar 7;23(3):115. doi: 10.3390/md23030115 (PMC11944246; doi:10.3390/md23030115)

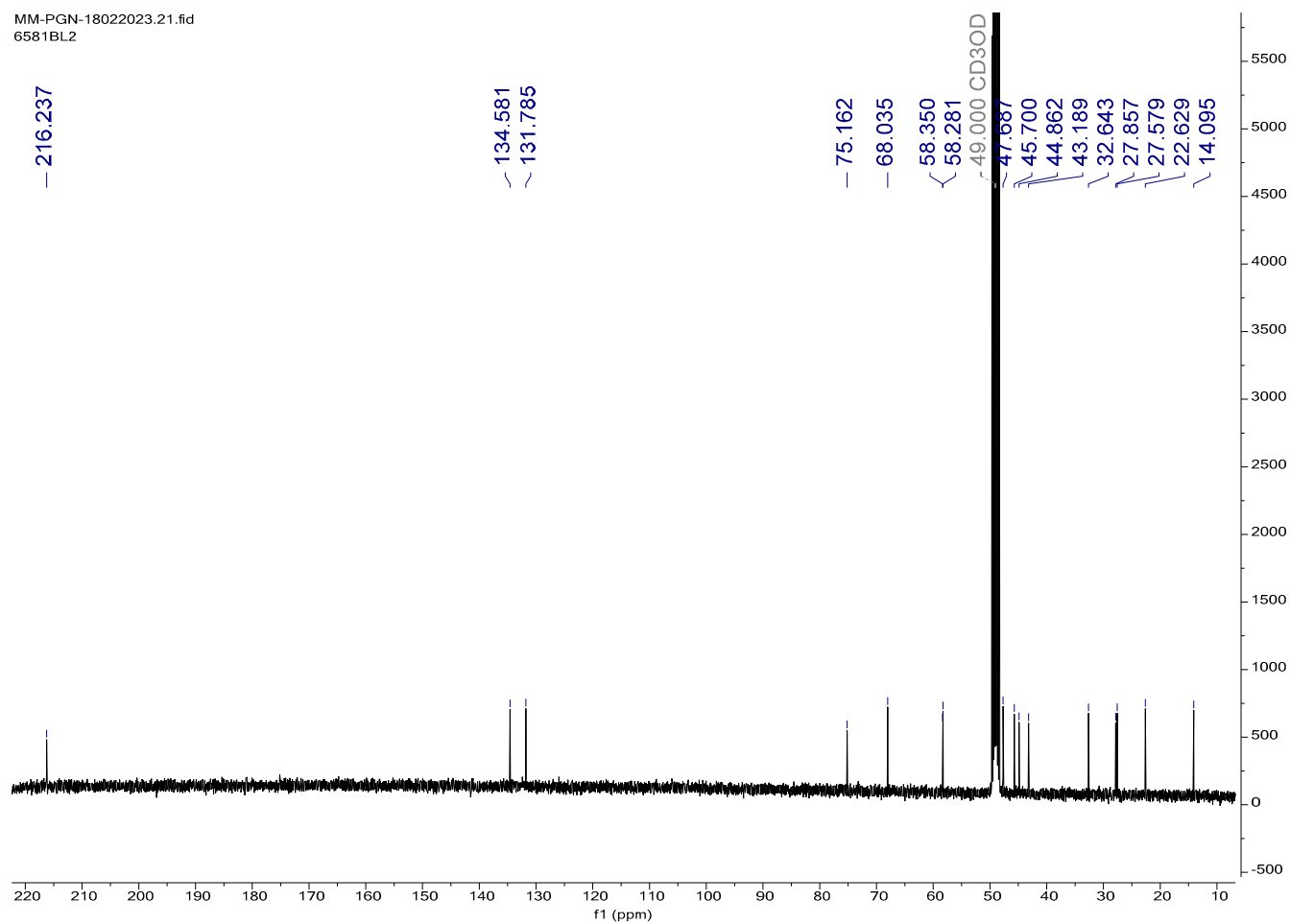

Figure S10. <sup>13</sup>C NMR (100 MHz) spectrum of 2 in CD<sub>3</sub>OD

Supplement: Supplementary file 1 [file marinedrugs-23-00115-s001.zip › Figure S10.pdf]

T: FTMS + p ESI Full ms [100.0000-1500.0000]

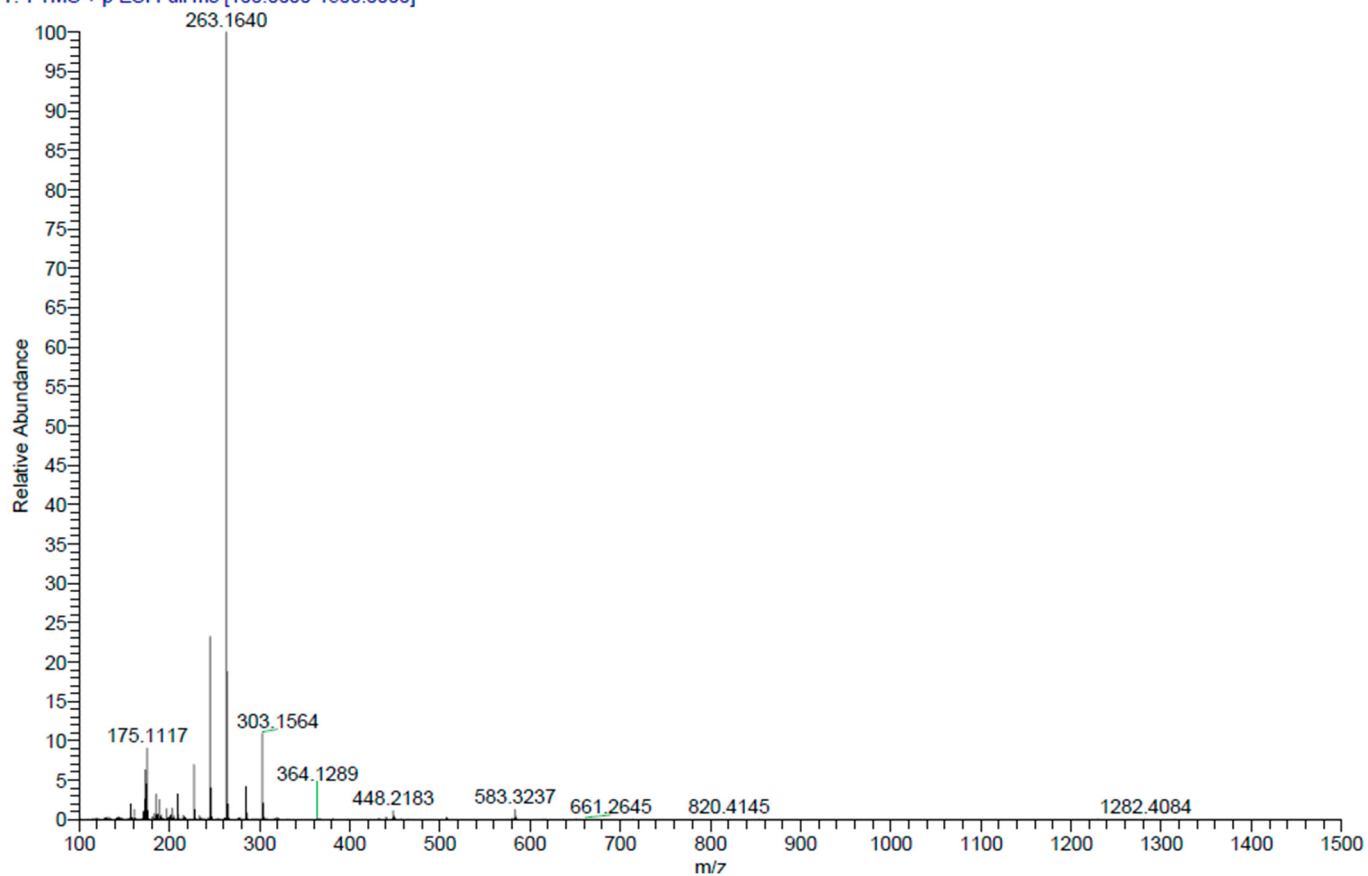

Figure S5. HRESIMS of 1

Supplement: Supplementary file 1 [file marinedrugs-23-00115-s001.zip › Figure S5.pdf]

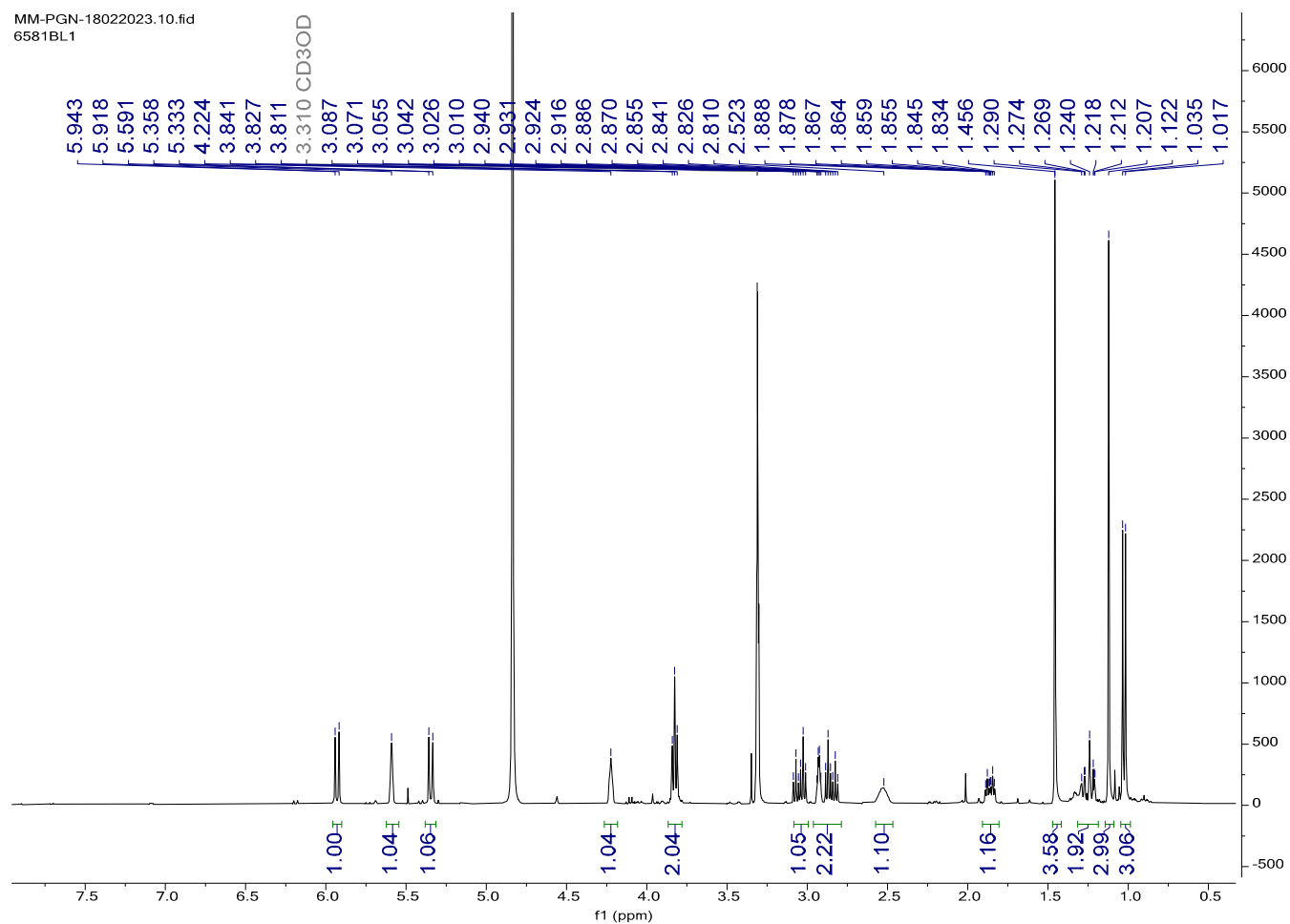

Figure S6. <sup>1</sup>H NMR (400 MHz) spectrum of **1** in CD<sub>3</sub>OD

Supplement: Supplementary file 1 [file marinedrugs-23-00115-s001.zip › Figure S6.pdf]

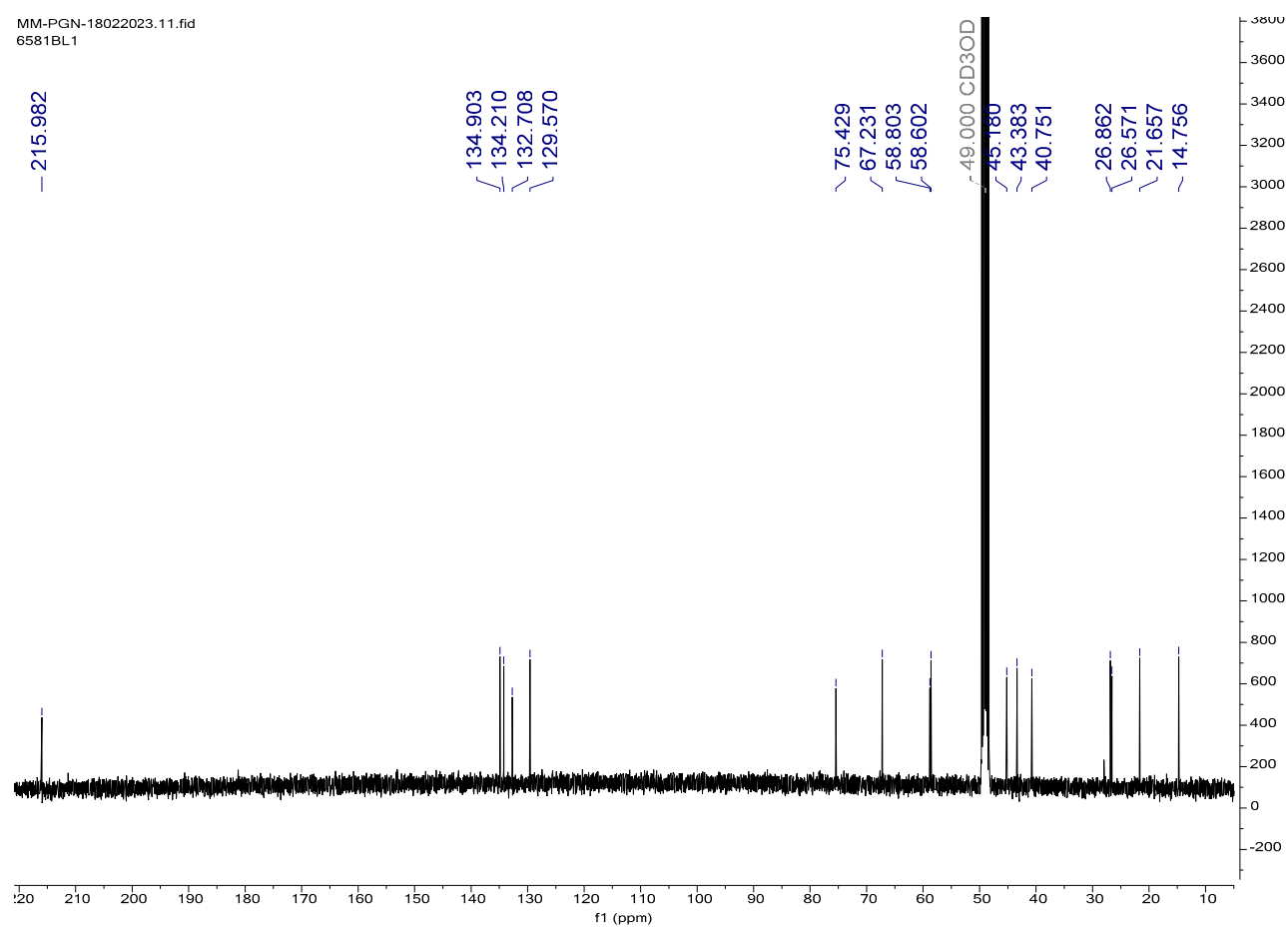

Figure S7. <sup>13</sup>C NMR (100 MHz) spectrum of 1 in CD<sub>3</sub>OD

Supplement: Supplementary file 1 [file marinedrugs-23-00115-s001.zip › Figure S7.pdf]

T: FTMS + p ESI Full ms [100.0000-1500.0000]

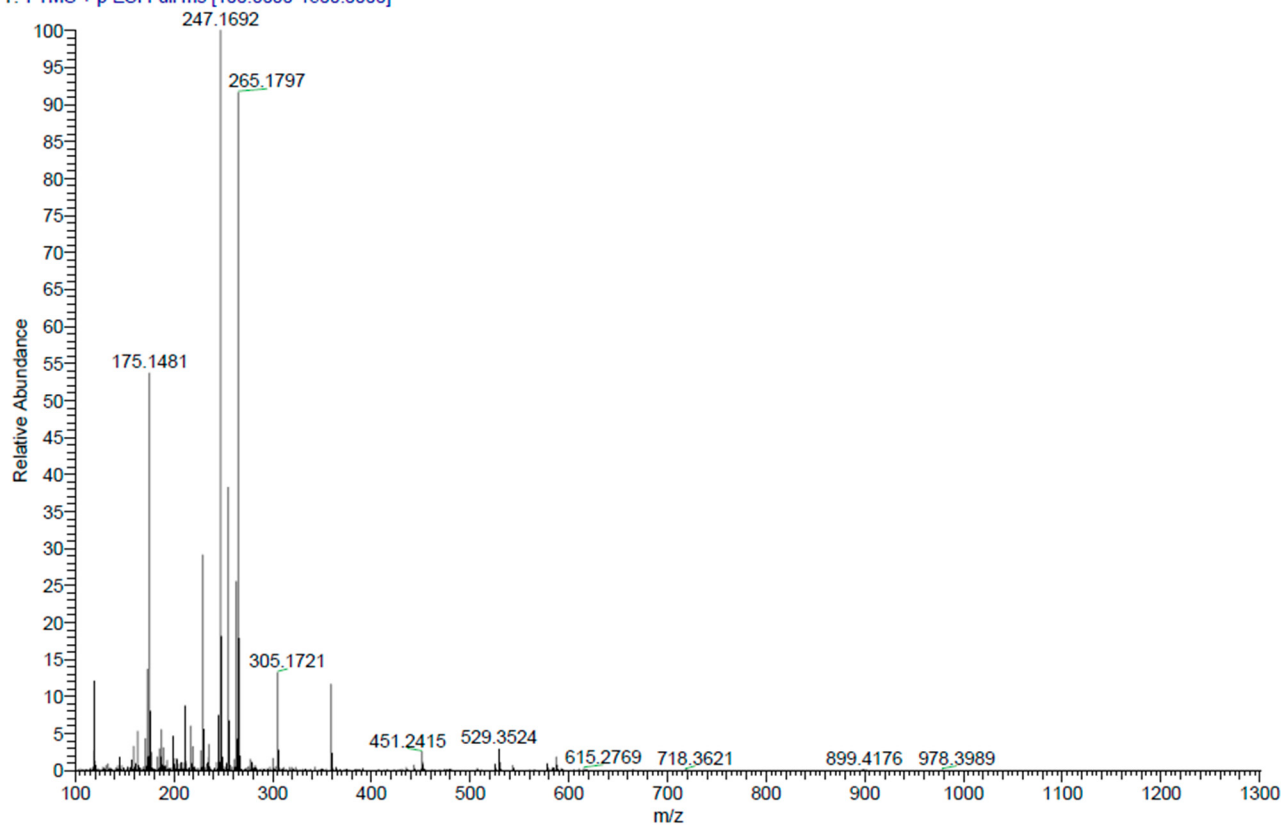

Figure S8. HRESIMS of 2

Supplement: Supplementary file 1 [file marinedrugs-23-00115-s001.zip › Figure S8.pdf]

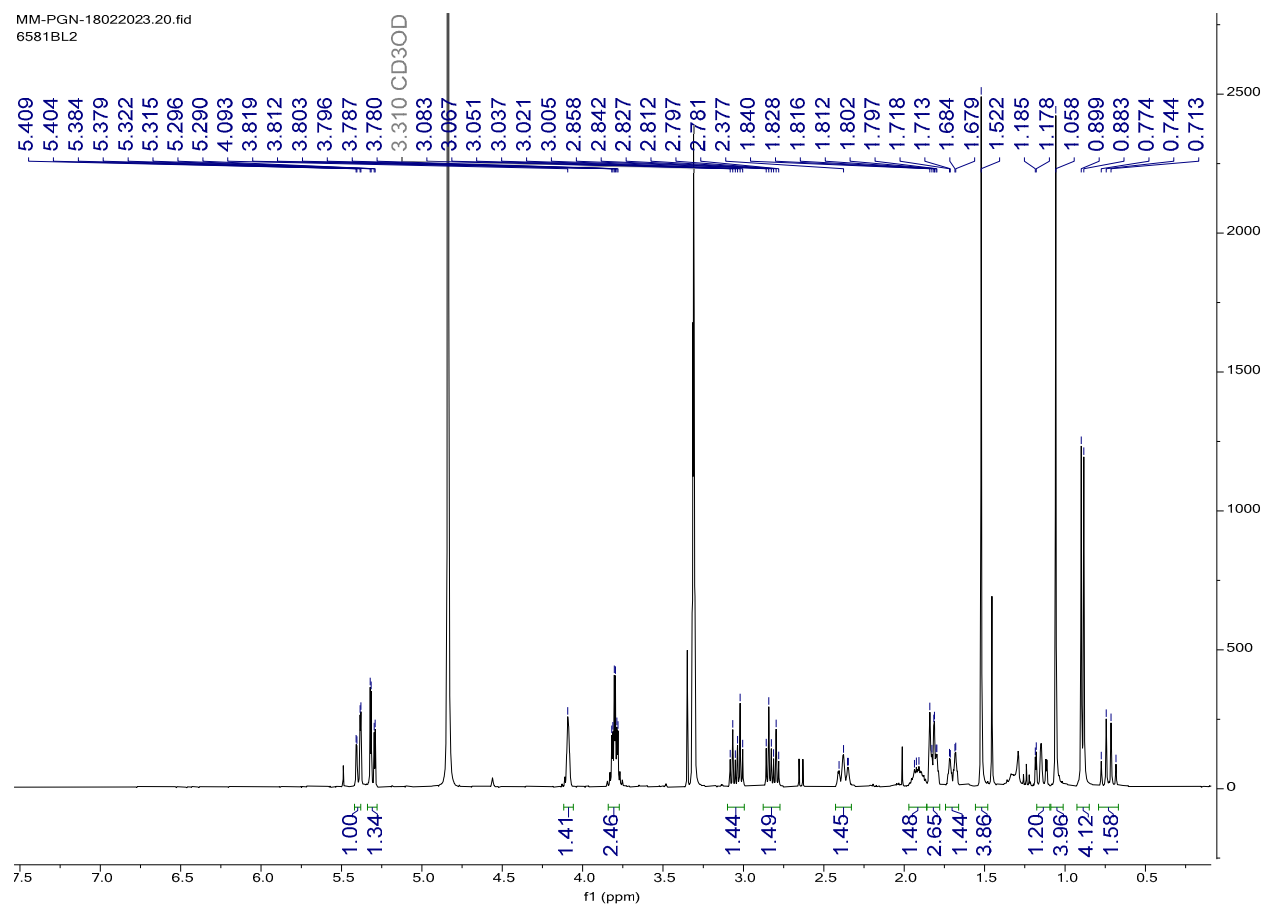

Figure S9. <sup>1</sup>H NMR (400 MHz) spectrum of 2 in CD<sub>3</sub>OD

Supplement: Supplementary file 1 [file marinedrugs-23-00115-s001.zip › Figure S9.pdf]
